# siRNAs and DNA Methylation Do a Two-Step to Silence Tandem Sequences

**DOI:** 10.1371/journal.pbio.0040407

**Published:** 2006-10-24

**Authors:** Richard Robinson

The genomes of higher organisms, including plants, are riddled with repetitive sequences, remnants of self-copying DNA parasites that randomly reinsert themselves, often harmlessly, but occasionally disrupting genes. Silencing these repeated elements is a major challenge for maintaining genomic health and is a major function of DNA methylation. In this process, a CH3 group is added onto one of the four DNA bases; groups of these altered bases reduce RNA polymerase’s access to the DNA, preventing transcription.

One common repeated element seen in genomes is tandem repeats, pairs of identical short DNA sequences lying next to each other. A longstanding question is how methylation machinery is directed to these tandem repeat sequences, which are frequently transcriptionally silenced. In a new study, Simon Chan, Steven Jacobsen, and colleagues show that both members of the pair are required, and their presence first stimulates production of small interfering RNAs (siRNAs). The siRNAs then attracts DNA methyltransferase, the enzyme directly responsible for methylation.

Only recently discovered, siRNAs have begun to pop up in many gene regulatory events. First transcribed as a larger RNA molecule, then diced into small fragments, siRNAs appear to control gene expression through multiple mechanisms. It has become clear that one of these mechanisms is the promotion of methylation—siRNAs have previously been found associated with methylated sites, and the authors recently showed that siRNAs could direct DNA methyltransferase to tandem repeats.

In *Arabidopsis*, the lab rat of the plant world, there are two tandem repeats near the beginning of a well-studied gene called *FWA* which are targeted for methylation. *FWA* is a good model for studying methylation, because when unmethylated *FWA* is inserted into *Arabidopsis*, 100% of the introduced genes become methylated, far more than other genes. When *FWA* is methylated, the plant flowers early. Mutants that leave the gene unmethylated flower late.

The authors first showed that the *FWA* tandem repeats are integral to triggering new methylation. An unmethylated *FWA* gene introduced into *Arabidopsis* plants that themselves had unmethylated *FWA* (and therefore flowered late) caused a portion of the transformed plants to flower early. This indicated that somehow the introduced gene triggered methylation of the endogenous *FWA* gene, as well as of itself (the unmethylated form is dominant, and so would stimulate late flowering unless it too had become methylated). When the tandem repeats were deleted from the introduced gene, the effect was lost. And when the tandem repeats alone, minus the rest of the gene, were introduced, the endogenous gene again became methylated and silenced, and flowering occurred early. Together, these results show the tandem repeats are both necessary and sufficient to stimulate methylation.

**Figure:**
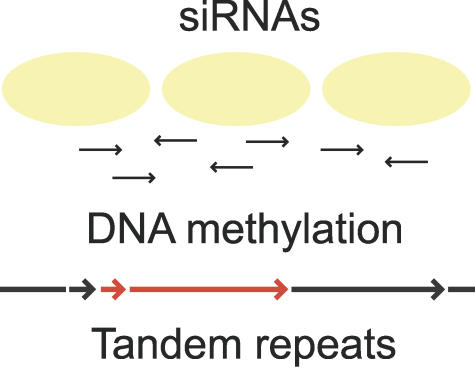
Tandem repeats recruit siRNA production and DNA methylation in two steps. (Image: Simon Chan)

To test whether it was the mere sequence of the repeats, or rather their double nature, that promoted methylation, the authors introduced a gene containing only one member of each tandem-repeat pair into plants with the nonmethylated form. No methylation took place, and the plants again flowered late. Thus, it appears that the sequences must be present as tandem repeats to direct methylation to *FWA*.

What was the mechanism by which the introduced gene triggered methylation? The authors have elsewhere shown that absence of any of the multiple factors responsible for synthesizing siRNA produces the same late-flowering phenotype, suggesting that siRNA is intimately connected to the methylation process. To find out, they examined the methylating ability of multiple plant lines, each a mutant for one or more genes in the siRNA methylation pathway, and also carefully measured the production of siRNAs complementary to the *FWA* tandem repeats (no mean feat, since their abundance is less than a tenth of one percent that of some other RNA species). They found that plants unable to methylate DNA could still produce siRNAs, while those that could not produce siRNAs could not carry out methylation. In addition, they made the surprising finding that siRNAs are produced from the unmethylated *FWA* gene. These findings show that siRNA-directed methylation is a two-step process, in which recruitment of siRNA production precedes recruitment of DNA methyltransferase.

Based on their results, the authors propose that tandem repeats act as attractors for the siRNA-making complex. Production of siRNA from these sequences then attracts the methylating machinery, leading to silencing of the gene containing the repeat. In this way, the gene regulatory apparatus functions somewhat like a genomic immune system, identifying potential threats and neutralizing them. As further evidence for this model, the authors showed that throughout the *Arabidopsis* genome, methylation of tandem repeats occurred at a much higher frequency when those repeats were associated with siRNAs than when they were not. Further exploration of this gene-silencing system may help explain how our genomes have been immunized against the ravages of parasitic DNA over life’s history, a process that continues into the present.

